# The Cost-Effectiveness of Early Access to HIV Services and Starting cART in the UK 1996–2008

**DOI:** 10.1371/journal.pone.0027830

**Published:** 2011-12-14

**Authors:** Eduard J. Beck, Sundhiya Mandalia, Roshni Sangha, Peter Sharott, Mike Youle, Guy Baily, Ray Brettle, Mark Gompels, Margaret Johnson, Brendan McCarron, Ed Ong, Anton Pozniak, Achim Schwenk, Stephen Taylor, John Walsh, Ed Wilkins, Ian Williams, Brian Gazzard, for the NPMS-HHC Steering Group

**Affiliations:** 1 NPMS-HHC CIC, Coordinating and Analytic Centre, London, United Kingdom; 2 London School of Hygiene & Tropical Medicine, London, United Kingdom; 3 Imperial College, London, United Kingdom; 4 London Specialised Commissioning Group, London Procurement Programme, London, United Kingdom; 5 Royal Free Hospital, London, United Kingdom; 6 Royal London and Barts Hospitals, London, United Kingdom; 7 Edinburgh General Hospital, Edinburgh, United Kingdom; 8 Southmead Hospital, Bristol, United Kingdom; 9 James Cook University Hospital, Middlesborough, United Kingdom; 10 Newcastle General Hospital, Newcastle, United Kingdom; 11 Chelsea and Westminster Hospital, London, United Kingdom; 12 North Middlesex Hospital, London, United Kingdom; 13 Birmingham Heartland Hospital, Birmingham, United Kingdom; 14 St. Mary's Hospital, London, United Kingdom; 15 North Manchester General Hospital, North Manchester, United Kingdom; 16 Mortimer Market Centre, London, United Kingdom; University Hospital Zurich, Switzerland

## Abstract

**Aim:**

To calculate use, cost and cost-effectiveness of people living with HIV (PLHIV) starting routine treatment and care before starting combination antiretroviral therapy (cART) and PLHIV starting first-line 2NRTIs+NNRTI or 2NRTIs+PI_boosted_, comparing PLHIV with CD4≤200 cells/mm3 and CD4>200 cells/mm3. Few studies have calculated the use, cost and cost-effectiveness of routine treatment and care before starting cART and starting cART above and below CD4 200 cells/mm3.

**Methods:**

Use, costs and cost-effectiveness were calculated for PLHIV in routine pre-cART and starting first-line cART, comparing CD4≤200 cells/mm3 with CD4>200 cells/mm3 (2008 UK prices).

**Results:**

cART naïve patients CD4≤200 cells/mm3 had an annual cost of £6,407 (95%CI £6,382 to £6,425) PPY compared with £2,758 (95%CI £2,752 to £2,761) PPY for those with CD4>200 cells/mm3; cost per life year gained of pre-cART treatment and care for those with CD4>200 cells/mm3 was £1,776 (cost-saving to £2,752). Annual cost for starting 2NRTIs+NNRTI or 2NRTIs+PI_boosted_ with CD4≤200 cells/mm3 was £12,812 (95%CI £12,685–£12,937) compared with £10,478 (95%CI £10,376–£10,581) for PLHIV with CD4>200 cells/mm3. Cost per additional life-year gained on first-line therapy for those with CD4>200 cells/mm3 was £4639 (£3,967 to £2,960).

**Conclusion:**

PLHIV starting to use HIV services before CD4≤200 cells/mm3 is cost-effective and enables them to be monitored so they start cART with a CD4>200 cells/mm3, which results in better outcomes and is cost-effective. However, 25% of PLHIV accessing services continue to present with CD4≤200 cells/mm3. This highlights the need to investigate the cost-effectiveness of testing and early treatment programs for key populations in the UK.

## Introduction

Recent studies have demonstrated the cost-effectiveness of starting combination antiretroviral therapy (cART) at CD4 counts between 201–350 cells/mm3 [1]. Analyses comparing specific treatment regimens demonstrated improved outcomes and lower costs when starting cART with higher CD4 counts; a CD4 count of 200 cells/mm3 remains a watershed cut-off point with those starting ART>200 CD4 cells/mm3 having better outcomes, using fewer services with lower costs compared with those starting ART≤200 cells/mm3 [1].

Most recent studies have focused on comparing different cART regimens. Few have recently investigated the use of services, their cost and cost-effectiveness of starting cART above or below a CD4 count of 200 cells/mm3; even fewer studies have estimated the use and cost of services by people living with HIV (PLHIV) before starting cART and the cost-effectiveness of this pre-cART treatment and care. If PLHIV are diagnosed with a CD4>200 and are linked to HIV services and followed up on a regular basis, this will enable them to start cART at a more optimum CD4 count.

The first objective of this study was to estimate the annual use, cost and cost-effectiveness of service provision for those PLHIV entering routine HIV treatment and care before starting cART, comparing those with CD4≤200 cells/mm3 with those with a CD4 count >200 cells/mm3; secondly to estimate the use, cost and cost-effectiveness of starting first-line with two nucleoside reverse transcriptase inhibitors with a non-nucleoside reverse transcriptase inhibitor or boosted protease inhibitor -2NRTIs+NNRTI or 2NRTIs+PI_boosted_ respectively - with a CD4≤200 cells/mm3 compared with a CD4>200 cells/mm3.

## Methods

The National Prospective Monitoring System on the use, cost and outcome of HIV service provision in UK hospitals - HIV Health-economics Collaboration (NPMS-HHC) has been monitoring prospectively the effectiveness, efficiency, equity and acceptability of treatment and care in participating HIV units since 1996. Using an agreed minimum dataset, standardized data are routinely collected in clinics and transferred to the NPMS-HHC Coordinating and Analytic Centre (CAC). Since the data were transferred in pseudo-anonymized format, patient consent was not required according to the UK Department of Health, which is in line with international guidelines [2]. While ensuring patient and clinic confidentiality, the data were analyzed at clinic and aggregate levels: clinic specific analyses remain confidential, while aggregate analyses become public documents [3], [4].

### Use and cost of services

Data on the use of hospital inpatient, outpatient and dayward services between 1^st^ January 1996 and 31^st^ December 2008, were obtained from computerized information systems from 15 UK hospitals participating in this analysis. 2NRTIs+NNRTI or 2NRTIs+PI_boosted_ regimens are currently the preferred regimens for starting first-line cART and have been routinely available in NPMS-HHC clinics since 1996. Subjects who started these regimens since 1996 were included in the study while patients who were transferred from other HIV units were excluded as it was not possible to establish with certainty whether these regimens were indeed their first-line regimen.

The mean numbers of inpatient days, outpatient visits and dayward visits per patient-year (PPY) were calculated for naïve patients before they started cART and those who started first-line 2NRTIs+NNRTI or 2NRTIs+PI_boosted_. A patient-year was defined as 365.25 days of follow up. The denominator for cART naïve patients consisted of the total duration of follow up for all cART naïve patients before starting cART, from when they were first seen until the end of the study period if still alive and cART naïve, or the data were censored at the date if they started cART, were lost to follow up or if they had died.

The denominator for those on first-line 2NRTIs+NNRTI or 2NRTIs+PI_boosted_ consisted of the total duration of follow up during the period of first-line treatment with 2NRTIs+NNRTI or 2NRTIs+PI_boosted_, from when they were first seen till the end of the study period if still alive, or the date if they failed first-line 2NRTIs+NNRTI or 2NRTIs+PI_boosted_, were lost to follow-up or died, which ever came first.

First-line 2NRTIs+NNRTI or 2NRTIs+PI_boosted_ failure was defined as any change made to the regimens, which included intensification of regimen by adding any anti-retroviral drug to the regimen or swapping the NNRTI or a PI_boosted_ to another anti-retroviral drug class. Dropping a NRTI, NNRTI or PI_boosted_ alone or simplification of anti-retroviral (ARV) combination with no other changes made to the regimen did not constitute treatment failure. Causes for failure included clinical, immunological or virological reasons and others, where adverse effects were the most likely cause [5].

Numerators for cART naïve patients were calculated by summing the use of inpatient, outpatient or dayward services until first-line cART, while numerators for those on first-line 2NRTIs+NNRTI or 2NRTIs+PI_boosted_ were calculated by summing the use of inpatient, outpatient or dayward services while on these first-line regimens. Mean use of services and 95% confidence intervals (95%CI) PPY were calculated using the Poisson test for the naïve population and those who started first-line 2NRTIs+NNRTI or 2NRTIs+PI_boosted_ disaggregated by CD4 count when starting routine treatment and care or first-line ART respectively. The mean use of services PPY was calculated based on a method for calculating the use of services employed in previous studies [1], [6], [7], [8] and summarized by the formula:
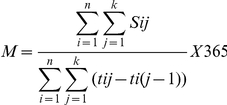
Where n = total number of individuals;

k = day of censoring;

S_ij_ = use of service of individual i at jth day;

t_ij_ = number of days starting and remaining cART naïve *or* on first-line 2NRTIs+NNRTI or 2NRTIs+PI_boosted_ by CD4 stratum for individual i;

M = mean of services S per patient-year by CD4 stratum.

The unit cost for an average inpatient day was £511, £101 for an outpatient visit and £413 per dayward visit [9]. Inpatient, outpatient and dayward costs PPY were obtained by multiplying their means and 95%CIs PPY by their respective unit costs for PLHIV starting at different CD4 counts. For naïve patients the costs generated by the use of services for each of the CD4 categories were added to the costs of ‘other’ drugs, tests and procedures performed; for those on first-line 2NRTIs+NNRTI or 2NRTIs+PI_boosted_, the use of services costs were added to the cost of ARVs, ‘other’ drugs and tests and procedures performed [9]. The costs for the different 2NRTIs+NNRTI or 2NRTIs+PI_boosted_ regimens were the weighted average annual prices based on prices negotiated by the London HIV Consortium in 2008 with pharmaceutical companies. Furthermore, the costs for ‘other drugs’ and test and procedures were weighted by stage of HIV infection: asymptomatic, symptomatic non-AIDS or AIDS. The study was performed from a public service perspective [10] and costs for use of services, ‘other’ drugs, tests and procedures performed, were obtained from the 2010 NPMS-HHC report [9]. Costs were calculated in UK pounds (2008 prices) and time-to-starting cART, time to first-line failure of 2NRTIs+NNRTI or 2NRTIs+PI_boosted_ regimens and treatment costs were discounted at 3.0% per annum [11].

### CD4 counts when starting pre-cART routine treatment and care and cART

Baseline CD4 counts were obtained within 4 months before or after of starting routine treatment and care for cART naïve patients and for those starting first-line 2NRTIs+NNRTI or 2NRTIs+PI_boosted_. In a minority of cases CD4 counts could not be obtained and for these patients their baseline CD4 count was imputed. For the cART naïve patients a total of 690 (2%) CD4 counts were imputed; for those starting 2NRTIs+NNRTI or 2NRTIs+PI_boosted_, 733 (12%) PLHIV had their CD4 count imputed.

CD4 counts were imputed using the Multiple Imputations (MI) procedure in SAS assuming that the data were multivariate normally distributed and that the missing CD4 count data were missing at random (MAR). The missing CD4 counts were substituted with an estimated value using a multiple imputation procedure which replaced each missing value with a set of plausible values that represented the uncertainty about the right value to impute [12]. The Markov chain Monte Carlo (MCMC) statement was used to predict mean matching method for imputation, a method that assumed multivariate normality [13]. The MCMC method imputed an observed value that was nearest to the predicted value from the simulated regression model for each missing value imputed.

### Regression Models, Time-to-starting cART and Time-to-Treatment Failure

Parametric quantitative data are presented as means with 95% confidence intervals (95%CIs) or standard deviation (SD) while non-parametric data are presented as medians with inter-quartile range (IQR). Between group comparisons of parametric data with more than two independent groups were tested using one-way-ANOVA while two independent groups were compared using unpaired t-test. Between group comparisons of non-parametric data with three independent groups were tested using the Kruskal-Wallis test while two independent groups were compared using the Mann-Whitney U test. Qualitative data by CD4 count strata were tested using the χ^2^ test and where appropriate these were adjusted using Yates' correction.

Median and inter-quartile ranges were used to create categories, including a separate category for all variables with missing data. This ensured no degrees of freedom were lost when building multivariable models. Cox's proportional hazards regression models with single variables were initially used to estimate likelihood of treatment failure. All variables found to have a probability of *p*<0.2 in univariate Cox's proportional hazards model were used to build a multivariable model to assess the risk of a particular prognostic variable while controlling for the other variables in the model.

The final multivariable models of the CD4 strata presented were tested for their distributional assumptions using Cox Snell residual plots and adjusted for sex, age, baseline viral load, baseline CD4 count, stage of HIV infection, other variables in the models and stratified by year of starting pre-cART routine treatment and care or first line 2NRTIs+NNRTI or 2NRTIs+PI_boosted_ for possible confounding or residual effects. Baseline viral load and CD4 cell count were defined as those available 4 months before or after starting pre-cART routine treatment and care or starting first-line 2NRTIs+NNRTI or 2NRTIs+PI_boosted_; baseline clinical stage was based on the diagnosis within 30 days since starting pre-cART routine treatment and care or 2NRTIs+NNRTI or 2NRTIs+PI_boosted_. Event time was defined as time-to-starting cART or time-to-treatment failure of first-line ART based on patient days of follow up. These were estimated from the start of the study period of 1^st^ January 1996, or the date of entry into the cohort if entry to the cohort came after this date, to either the end of the study period of 31^st^ December 2008 or starting cART, failure of 2NRTIs+NNRTI or 2NRTIs+PI_boosted_ regimens, or the last recorded visit during their follow-up.

### Survival Function Estimation

After adjusting for confounding and residual variables in the final model, the PROC PHREG in SAS was run with the BASELINE statement to create a new data set with the “survival” function estimates at the event times of each stratum for each list of variables in the final multivariable model [14]. This contained the “survival” function estimates corresponding to the means of the variables in the model for each stratum. The resulting survival function estimates were used to model with event time as a covariate based on the least squares maximum likelihood model. The resulting least squares regression model was then used to estimate the extrapolated median and inter quartile ranges (IQR) of time-to-starting cART or first-line treatment failure. All analyses were performed using SAS version 9.1.3 statistical software and all significance tests presented are two-tailed.

### Life year gained for cART naïve patients and those on first-line cART

Based on differences in the estimated starting or failure times, the additional life years gained in routine treatment and care of pre-cART or on first-line 2NTRIs+NNRTI or 2NRTIs+PI_boosted_ regimens by CD4 stratum were calculated based on methods used for previous analyses [1], [6], [7], [8]. The incremental cost-effectiveness ratios (ICERs) were calculated using time-to-starting cART or time-to first-line 2NRTIs+NNRTI or 2NRTIs+PI_boosted_ failure respectively as the outcome measures of the two analyses and were calculated using the following formula [10]:
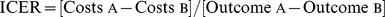
A cost-effectiveness analysis and scenario analyses were produced for the cART naïve population and those that started first-line 2NTRIs+NNRTI or 2NRTIs+PI_boosted_. The lower bound of the scenatio analyses consisted of the lower 95% confidence interval of the annual cost combined with the lower quartile of time-to-starting cART or time-to-first line failure of 2NTRIs+NNRTI or 2NRTIs+PI_boosted_ regimens respectively; the upper bound consisted of the upper 95% confidence interval of the annual cost combined with upper quartile of time-to-starting ART or time-to-first line failure of 2NTRIs+NNRTI or 2NRTIs+PI_boosted_ regimens respectively.

## Results

### cART naïve patients

**Figure 1:**
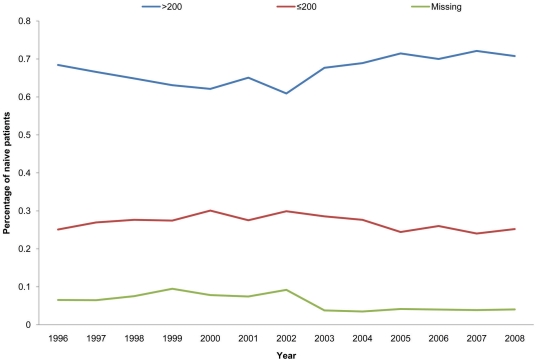
Proportion of cART naïve PLHIV diagnosed and entered routine treatment and care with CD4≤200 cells/mm3, CD4>200 cells/mm3 and missing CD4 count, 1996–2008.

A total of 30,558 naïve patients were seen during the study period in participating clinics. Of these 8,155 (27%) had a CD4≤200 cells/mm3 when first starting pre-cART routine treatment and care, 20,496 (67%) had a CD4 count >200 cells/mm3 and 1,907 (6%) did not have a CD4 recorded nor could this be imputed. The proportion of PLHIV who were first diagnosed with a CD4≤200 cells/mm3 remained remarkably constant during the study period at around 25% (Figure 1). Patients presenting with a CD4≤200 cells/mm3 were more likely to be older, women or black Africans, and attend a non-London clinic compared with those presenting with a CD4 count >200 cells/mm3. Mean baseline CD4 count for the former group was 64 cells/mm3 (95% CI 62–65) compared with 439 cells/mm3 (95%CI 436–441) for the latter (Table 1).

**Table 1:** Demographic characteristics, mean (95%CI) use of services and associated annual costs for cART naïve people living with HIV by CD4 strata.

	CD4≤200 N = 8155 (27%)	CD4>200 N = 20496 (67%)	CD4 Missing N = 1907 (6%)	p-value
Sex				<0.001
Female	2267 (27.8)	3994 (19.5)	406 (21.3)	
Male	5888 (72.2)	16502 (80.5)	1501 (78.7)	
Mean Age (SD)	35.7 (10.5)	32.6 (9.1)	33.5 (9.7)	<0.001
Ethnic group				<0.001
Unknown	1606 (19.7)	2926 (14.3)	475 (24.9)	
Other	1325 (16.2)	3274 (16.0)	261 (13.7)	
Black African	1896 (23.2)	2641 (12.9)	334 (17.5)	
Caucasian	3328 (40.8)	11655 (56.9)	837 (43.9)	
IDU				<0.001
Yes	396 (4.9)	926 (4.5)	183 (9.6)	
No	7759 (95.1)	19570 (95.5)	1724 (90.4)	
Geometric mean CD4 count (95%CI) cells/mm3	64 (62–65)	439 (436–441)	Not applicable	<0.001
Clinic location			Not applicable	<0.001
London	6,054 (26.6)	16,692 (73.4)		
Non-London	2,101 (35.6)	3,804 (64.4)		

	CD4≤200	CD4>200
Mean Inpatient		
Days (95% CI)	2.76 (2.74–2.78)	0.41 (0.40–0.42)
Costs	£1411 (£1398–£1422)	£207 (£205–£208)
Mean Outpatient		
Visits (95% CI)	8.32 (8.25–8.34)	6.22 (6.21–6.23)
Costs	£840 (£832–£842)	£628 (£627–£629)
Mean Dayward		
Visits (95% CI)	0.77 (0.76–0.78)	0.41 (0.40–0.42)
Costs	£318 (£312–£323)	£168 (£166–£170)
Average use of services costs – subtotal (95% CI)	£2,569 (£2,544–£2,587)	£1,004 (£998–£1,007)
Other drug cost	£3,070	£1,293
Tests + procedures	£768	£461
**Total annual cost (95% CI)**	**£6,407 (£6,382–£6,425)**	**£2,758 (£2,752–£2,761)**

Use of inpatient, outpatient and dayward services were significantly higher in those patients with CD4≤200 cells/mm3 resulting in higher costs annual costs of services of £2,569 (95%CI £2,544 to £2,587) compared with £1,004 (95% CI £998 to £1,007) for cART naïve patients presenting with CD4 count >200 cells/mm3. ‘Other’ drug cost and costs for tests and procedures were also higher for the naïve patients who presented with CD4≤200 cells/mm3, resulting in a substantially higher total annual cost of £6,407 PPY (95%CI £6,382 to £6,425) for these patients compared with £2,758 PPY (95%CI £2,752 to £2,761) for PLHIV with CD4>200 cells/mm3 (Table 1).

Estimated time to starting ART was 0.7 years (IQR 0 to 5.6) for those presenting with a CD4≤200 cells/mm3 compared with 5.3 years (IQR 1.0 to 9.8) for those presenting with CD4>200 cells/mm3 (Table 2). This resulted in a cost of £1,776 per life year gained of routine treatment and care pre-cART, with a lower bound of £2,752 and an upper bound which was cost-saving (−£3,615) per additional life-year gained in pre-cART treatment and care (Table 3)

**Table 2:** Multivariable Cox's proportional hazards regression model showing significant independent predictors of the likelihood of starting first-line cART.

		*Hazard Ratio	95% CI	Score statistics p-value
Age at HIV diagnosis	Missing	1.12	(1.06–1.18)	<0.001
	≤27.2	0.95	(0.90–1.00)	0.044
	27.3–32.5	0.99	(0.94–1.04)	0.579
	32.6–38.5	1.00	(0.95–1.05)	0.886
	>38.5	1		
Sex	Female	1.23	(1.19–1.28)	<0.001
	Male	1		
First ever viral load (missing imputed)	Missing	1.15	(0.90–1.48)	0.269
	>144000	1.57	(1.50–1.65)	<0.001
	29201–144000	1.40	(1.33–1.46)	<0.001
	3601–29200	1.01	(0.96–1.06)	0.682
	≤3600	1		
First Diagnosis	AIDS	1.42	(1.37–1.48)	<0.001
	Non AIDS	1		
First ever CD4	Missing	0.15	(0.11–0.20)	<0.001
	>200	0.53	(0.51–0.55)	<0.001
	≤200	1		

*Adjusted for sex, baseline VL, Baseline AIDS, and stratified by year of starting pre-cART routine treatment and care and other variables in the model.

**Table 3:** Estimated time-to-starting first-line cART and cost-effectiveness of starting pre-cART routine treatment and care, comparing PLHIV starting with CD4>200 cells/mm3 and CD4≤200 cells/mm3 (lower and upper bounds).

PLHIV starting pre-cART routine treatment and care		
	≤200 CD4 cells/mm3	>200 CD4 cells/mm3
Estimated median (IQR) days to starting cART	242 (0 to 2028)	1941 (355 to 3527)
**Cost – effectiveness of additional year gained as naïve patient (lower – upper bound)**	**£1776 [£2752 to cost-saving (−£3615)]**	

### Patients starting antiretroviral therapy

**Figure 2:**
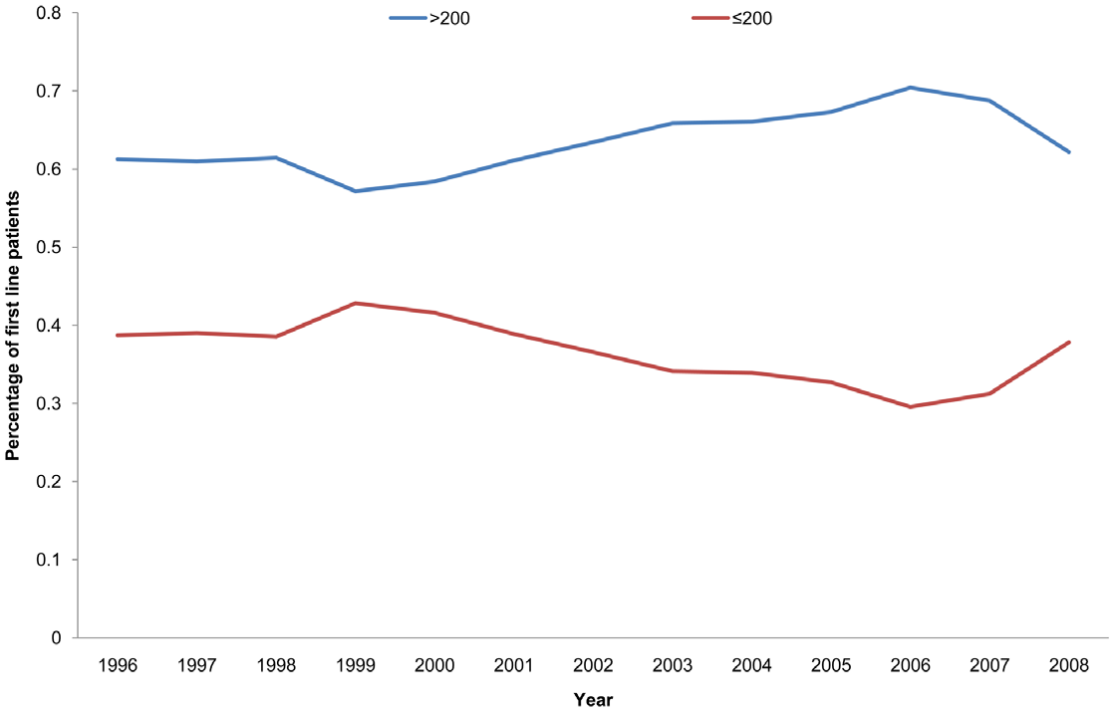
Proportion of PLHIV starting 2NTRIs+NNRTI or 2NRTIs+PI_boosted_ with CD4≤200 cells/mm3 and CD4>200 cells/mm3, 1996–2008.

During the study period, 5,858 patients started on 2NTRIs+NNRTI or 2NRTIs+PI_boosted_ regimens. Of these, 2,134 (36%) started with a CD4≤200 cells/mm3, and the remaining 3,724 (64%) started with CD4>200 cells/mm3. Again, these proportions remained relatively stable during the study period (Figure 2). Most of the demographic characteristics of those who started cART were similar to the cART naïve patients (Table 4). PLHIV starting cART with CD4≤200 cells/mm3 were older, more likely to be women or black African, however they were also more likely to have an history of injecting drugs; no differences were observed whether they were being managed in clinics in- or outside of London. The mean CD4 count differed significantly between the groups and was 83 cells/mm3 (95% CI 79–86) compared with 350 cells/mm3 (95%CI 346–354).

**Table 4:** Demographic characteristics, mean (95%CI) use of services and associated annual costs for people living with HIV on first-line 2NTRIs+NNRTI or 2NRTIs+PI_boosted_ by CD4 strata.

	CD4≤200N = 2134 (%)	CD4>200N = 3724 (%)	p-value
Sex			<0.001
Female	578 (27.1)	816 (21.9)	
Male	1556 (72.9)	2908 (78.1)	
Mean Age (SD)	38.0 (9.3)	36.8 (9.4)	<0.001
Ethnic group			<0.001
Unknown	311 (14.6)	386 (10.4)	
Other	354 (16.6)	537 (14.4)	
Black African	498 (23.3)	637 (17.1)	
Caucasian	971 (45.5)	2164 (58.1)	
IDU			0.016
Yes	109 (5.1)	141 (3.8)	
No	2025 (94.9)	3583 (96.2)	
Geometric mean CD4 count (95%CI) cells/mm3	83 (79–86)	350 (346–354)	p<0.001
Clinic location			p = 0.112
London	1,519 (35.8)	2,722 (64.2)	
Non-London	615 (38.0)	1,002 (62.0)	

	CD4≤200	CD4>200
Mean Inpatient		
Days (95% CI)	2.94 (2.89–2.99)	1.38 (1.35–1.41)
Costs	£1501 (£1474–£1529)	£705 (£691–£719)
Mean Outpatient		
Visits (95% CI)	7.88 (7.78–7.96)	7.12 (7.05–7.20)
Costs	£795 (£786–£804)	£719 (£712–£727)
Mean Dayward		
Visits (95% CI)	1.23 (1.20–1.27)	0.82 (0.80–0.84)
Costs	£510 (£495–£523)	£339 (£330–£348)
Average use of services costs – subtotal (95% CI)	£2,806 (£2,755–£2,856)	£1,763 (£1,733–£1,794)
Average annual cART costs (95% CI)	£5,998 (£5,923–£6,074)	£6,166 (£6,094–£6,238)
Other drug cost	£3,214	£1,971
Tests + procedures	£793	£578
**Total Annual cost (95% CI)**	**£12,812 (£12,685–£12,937)**	**£10,478 (£10,376–£10,581)**

Use of inpatient, outpatient and dayward services was significantly greater in those who started with a CD4≤200 cells/mm3 and this was reflected in higher annual costs of £2,806 (95%CI £2,755–£2,856) compared with £1,763 (95% CI £1,733–£1,794) for those starting cART with CD4>200 cells/mm3. Cost of first-line cART was similar for both groups, while the group starting cART with lower CD4 counts had higher ‘other’ drug costs and costs for tests and procedures (Table 4). The total annual cost for the group starting with CD4≤200 cells/mm3 was £12,812 PPY (95%CI £12,685–£12,937), which was significantly higher compared with the group starting with CD4>200 cells/mm3 which had an annual cost of £10,478 PPY (95%CI £10,376–£10,581; Table 4).

The estimated time to first-line treatment failure was 14.7 years (IQR 6.8 to 22.7) for those starting with a CD4>200 cells/mm3 compared with 10.4 years (IQR 4.8–16.0) for those who started with a CD4≤200 cells/mm3 (Table 5). The average cost per additional life-year gained on first-line therapy for those starting cART with CD4>200 cells/mm3 was £4639, with a lower bound of £3,967 and upper bound of £2,960 per additional life year gained on first-line cART (Table 6).

**Table 5:** Multivariable Cox's proportional hazards regression model showing significant independent predictors of the likelihood of treatment failure for first-line 2NTRIs+NNRTI or 2NRTIs+PI_boosted_.

		*Hazard Ratio	95% CI	Wald statistics p-value
Age at start of therapy	≤31.3	1.47	(1.18–1.84)	<0.001
	31.4–36.5	1.60	(1.30–1.98)	<0.001
	36.6–42.3	1.52	(1.22–1.88)	<0.001
	>42.3	1		
Baseline CD4 range (missing imputed)	≤200	1.23	(1.06–1.43)	0.007
	>200	1		
ART at start of 1^st^ line HAART	2NA+PI_boosted_	1.12	(0.93–1.36)	0.227
	2NA+NNRTI	1		

*Adjusted for sex, baseline VL, Baseline AIDS, and stratified by year of starting first-line 2NTRIs+NNRTI or 2NRTIs+PI_boosted_ and other variables in the model.

**Table 6:** Estimated time-to-first-line 2NTRIs+NNRTI or 2NRTIs+PI_boosted_ treatment failure and cost-effectiveness of starting 2NTRIs+NNRTI or 2NRTIs+PI_boosted_, comparing PLHIV starting with CD4>200 cells/mm3 and CD4≤200 cells/mm3 (lower and upper bounds).

2NTRIs+NNRTI or 2NRTIs+PI_boosted_		
	≤200 CD4 cells/mm3	>200 CD4 cells/mm3
Estimated median number of days (IQR) to first line failure for those who started cART (p = 0.007)	3805 (1753 to 5857)	5376(2483 to 8269)
**Cost – effectiveness LYG greater versus less/equal 200 cells/mm3 CD4 count (lower – upper bound)**	**£4639 (£3967 to £2960)**	

## Discussion

For PLHIV to be diagnosed early after they have been infected and have access to HIV services is to ensure that they can be monitored over time and start cART at an optimum CD4 count, when the immunological damage inflicted by HIV infection is less extensive and response to cART is optimal. Given that the additional costs per life-year gained are well below the £35,000 cut-off point, at which NICE considers interventions not to be cost-effective [15], providing access to HIV services for PLHIV with a CD4 count >200 cells/mm3 and monitoring them before they start cART is very cost-effective. It enables the immunological status of PLHIV to be monitored over time to ensure that these PLHIV start cART with CD4 counts above 200 cells/mm3.

Also remarkable was the considerable reduction in annual cost for both cART groups compared with earlier analyses [1]. This was due to a reduction in use of services but also a reduction in the ARV costs, which decreased over time, and points to the effectiveness of these regimens. These are some of the efficiencies which health services need to achieve to make service provision sustainable, especially during times of a global economic downturn. It does raise the question whether more community services are being used by these PLHIV and if so which services, provided by whom and their cost, cost-effectiveness and acceptability.

Current BHIVA and WHO guidelines advocate that PLHIV should start cART when CD4 count drops below 350 cells/mm3 [16], [17]; other guidelines now suggest that cART can be started when CD4 counts drops below 500 cells/mm3 [18], while some practitioners have now started prescribing cART as soon as an individual has been diagnosed with HIV infection, irrespective of CD4 count [19]. While the START trial - due to end in 2015 - will provide answers to these questions [20], most practitioners agree that cART should be started well before CD4 counts drop below 200 cells/mm3.

Given the recent results of the HPTN 052 trial [21], the benefits for starting cART not only include increased survival and reduced morbidity of those PLHIV on cART, but the demonstrated reduction in infectivity will further contribute to the reduction in HIV transmission at individual and population levels. As was recently pointed out, any successful response to the UK HIV epidemic will need to include a reduction in HIV transmission through more effective HIV prevention measures, which are likely to require behavioural, biomedical and structural interventions [22].

Despite the large number of subjects followed-up over the years, the analyses as presented have their limitations. Firstly, the data were collected in 15 sites, including both London and out-of London clinics, but 79% of patients contributing to this study, were seen in London clinics. Secondly first CD4 count when starting routine treatment and care pre-cART or starting 2NTRIs+NNRTI or 2NRTIs+PI_boosted_ could not be retrieved for a small number of subjects and these had to have their CD4 count imputed. Thirdly, sub-analyses were not performed for specific groups of PLHIVs, like men who have sex with men (MSM) or specific ethnic groups and different populations may be diagnosed with different CD4 counts. Fourthly, the data available for operational research are by definition observational data [23]. While adjustments were made for some key potential confounders, some residual confounding may have remained and affected the results.

It remains disconcerting, that a quarter of PLHIV when first diagnosed and enrolled in these clinics for pre-cART services had a CD4≤200 cells/mm3, a proportion which remained constant during the study period. Recent studies performed in the UK highlighted the fact that different populations have been diagnosed with different CD4 counts. For instance, between 1993 and 2002, the percentage of MSM diagnosed with HIV and a CD4 count <200 cells/mm3 in England and Wales decreased from 38% to 25% [24]. For newly diagnosed heterosexual PLHIVs in England and Wales between 2000 and 2004, 42% had a CD4<200 cells/mm3 [25]. In the latter study, 74% of the heterosexual sample were black-Africans and only 11% Caucasian heterosexuals; 43% of black-African heterosexual individuals were diagnosed with a CD4 count <200 cells/mm3, compared with 36% of black-Caribbean or white heterosexual individuals respectively. Of the black-African heterosexuals infected in the UK, 20% were diagnosed with a CD4 count <200 cells/mm3 compared with 44% of heterosexuals infected in Africa [25].

As was recently reported [26], significant proportions of PLHIVs across Europe continue to present late and the authors of the study concluded that the “evidence points to high rates of late diagnosis across Europe – between 15% and 38% of all HIV cases – and concur that trends are increasing or at best stagnant” [26].

The implications for those presenting with CD4 counts ≤200 cells/mm3 is that once they start cART their estimated time-to-first-line treatment failure is significantly shorter that those who start with a CD4 count >200 cells/mm3, while the costs of services before and after starting cART are higher. In fact, of the estimated 83,000 individuals living with HIV in the UK in 2008 – which increased to an estimated 86,500 in 2009 - 26% were thought to be unaware of being infected and did not know their sero-status [27].

Whether it is cost-effective to institute testing and early treatment programs in the UK, either for key or general populations as was recently proposed in the US [28] remains to be assessed. However, as the US study reiterated, while early treatment can play an important part in containing a country's HIV epidemic, it is likely that only through combined behavioural, biomedical and structural interventions that the greatest impact can be achieved.
